# Introduction

**DOI:** 10.1177/10245294221082243

**Published:** 2022-04-17

**Authors:** Kean Birch, Callum Ward, Eliot Tretter

**Affiliations:** 7991 York University, Toronto, ON, USA; 4905 The London School of Economics and Political Science, London, UK; 2129 University of Calgary, Calgary, Canada

**Keywords:** Rentiership, economic rents, technoscientific capitalism, intellectual property, big tech, technoscience

## Introduction

In this guest introduction, we explore emerging critiques at the frontiers of *techno-economic rentiership*. Rentiership is a process entailing political-economic and technological (i.e. techno-economic) relations, formations, practices, and justifications underpinning the ownership and/or control of assets, which enable the capture of future revenue streams (i.e. what we call rents). Conceptually, in this context, rentiership permits us to analyse the increasingly diverse techno-economic dimensions of those future revenue streams, rather than being a normative term for identifying ‘good’ or ‘bad’ forms of revenue (i.e. profit vs. rent).

The late Anne Haila (1990, 2016) noted that theoretical interest in ‘rentiership’ often emerges in response to specific societal crises. Recently, there has been a notable resurgence in use of the concept of ‘economic rent’ in mainstream public and policy debates, especially among high profile progressive economists (e.g. Stiglitz, 2012; Piketty, 2014; Mazzucato, 2018). Despite having similar concerns, however, their understandings of rent are difficult to reconcile. Stiglitz (2012) lays considerable stress on the role of ‘rent seeking’ as the cause of social problems like inequality, where rent-seeking is defined as the exploitation of political processes (e.g. regulatory capture). Piketty's (2014) more expansive concept of rent refers to income on capital where revenue ‘is simply remuneration for *ownership* of the asset, independent of any labor’ (p. 422, our emphasis), while ‘undue or unjustified’ monopoly rents are the result of ‘imperfection in the market’ (p. 423). Finally, focusing on normative understandings of what counts as productive investment and what as rent-seeking (see also McGoey, 2017), Mazzucato (2018) argues that some economic activities should be reclassified from productive to non-productive (like rent on money) and others vice versa (such as a government services).

These progressive accounts continue to conceptualize rents through the prism of mainstream economics and so do little to theoretically advance earlier discussions of economic rent. Yet, recent scholarship, like the collection of papers here, argue that something is different today. In particular, monopolistic ‘Big Tech’ firms have been able to extend rentiership into non-traditional spheres through new and reinvigorated mechanisms of rent extraction, such as ‘platform’ business models or knowledge and data monopolies (Srnicek, 2016; Fields, 2019; Sadowski, 2020; Birch and Cochrane, 2021; Birch et al. 2021; Komljenovic, 2021; Rikap, 2021a). Previously, it was assumed that techno-economic rents were eroded by the competition mechanism (see Schumpeter, 1950; Mandel, 1975; Storper, 1997), but the combination of platform control over innovation networks and centrality of intellectual property in political-economic restructuring is forcing scholarship to reimagine the crucial question of whether this mechanism still holds (see Wark, 2019; Zacares, 2021).

The papers in this Special Section all contribute to conceptualizing these new forms and mechanisms of rentiership emerging from the increasing technoscientific nature of capitalism, as well as the modes of social conflict this engenders (see Zeller, 2008; Pagano and Rossi, 2009; Moulier Boutang, 2011; Vercellone, 2013; Felli, 2014; Frase, 2016; McGoey, 2017, 2021; Birch, 2020; Rikap and Lundvall, 2020; Birch and Cochrane, 2021; Komljenovic, 2021; MacKenzie, 2021; Rikap, 2021a; Strauss, 2021). Taken together, they are a significant contribution to expanding how we understand the frontiers of techno-economic rentiership and the socio-political responses these transformations are provoking.

### A Very Short History of Economic Rent and Technological Innovation

Conventional historiographies of economic rent often start with Adam Smith’s (1776) engagement with the Physiocrats. Smith defined ‘rent’ as a distinct form of revenue associated with land ownership and functionally separate from ordinary profits or wages. This ‘classical’ perspective focused on control of agrarian land as the key problematic of rentiership, reflected in the work of 19^th^ century political economists. Ricardo (1817 [2001]) was similarly concerned with profits from land ownership but criticized Smith’s focus on the absolute relationship, developing the principle of *differential* rent to reflect the qualitative differences in land. More productive and fertile land enabled owners to command a relatively higher return and this differential profit was captured by the landowner. Later, Karl Marx (1894) integrated absolute and relative forms of rent into his framework of socially necessary labour time. He conceptualized rentiership as the ability to capture the advances over the average rate of profits on agricultural lands (as opposed to the Ricardian focus on the least productive land in production).

Land was the focus of these political economists, but later thinkers began to associate rentiership with the rise of large corporate organizations and their role in developing and controlling new technologies. Early marginalists like Alfred Marshall (1890), for example, extended the concept of rent to explicitly include the value of machines. In his *Principles of Economics*, Marshall used ‘*quasi-rent*’ as a term ‘for the income derived from the machines and other appliances for production made by man’ (p. 51). According to Marshall, quasi-rents were derived from the ownership over the future value of a technology – or even animals like ponies – where these future earnings were specifically discounted (Marshall, 1890).

Thorstein Veblen (1904) also emphasized this pecuniary notion of rent in his institutional analyses of business enterprise. Veblen argued that rents are the result of institutional constraints that enable firms to exploit monopoly positions as price makers, rather than price takers, and thereby fix prices higher than what would be taken if there was ‘normal’ competition. Or, as Veblen (1904: 35) put it, ‘in the language of the railroads as “charging what the traffic will bear”’. Significantly, Veblen also stressed the role of intangible assets – capitalized property – in underpinning new kinds of rentiership – specifically resulting from ‘good-will, trademarks, brands, etc.’. Veblen was less concerned with the relationship between rentiership and the processes of technological innovation, however, other than that these can be important factors for how rentiership can be established by legal barriers.

In contrast to Marshall’s efforts to explain away rents or Veblen’s focus on extra-economic institutional control, Joseph Schumpeter, one of the most significant writers on the topic, essentially glorified the practice of rentiership. For Schumpeter (1950), the heroic role of the entrepreneur was to innovate, and he characterized the results of innovation as the most important and unique sources of growth in capitalism. Schumpeter was not concerned about the problematic side-effects of ‘entrepreneurial profits’ (i.e. rents), but argued that such rents were the just deserts for entrepreneurial ingenuity. Moreover, Schumpeter believed that imitation and diffusion of technologies would undermine these rents, making them only temporary. Significantly, Schumpeter predicted that entrepreneurial profits would be of declining significance to capitalist development because the growth of large-scale enterprises and state socialism would stymie the need for incentives for new innovations (ibid.).

Rent was also being used in neo-Marxist literature. Like Schumpeter, for example, Ernest Mandel (1975) stressed the crucial role of technological innovation in capitalism. However, Mandel did not believe that innovation generated any additional value; rather, it had an intermediate role in redistributing surplus-value. For Mandel, firms adopt new technologies because of their positive impact on their relative productivity rates (i.e. the relative rate of difference in production). Mandel believed innovation is a way that firms more extensively extract surplus-value from *within* their specific sphere of production. He called this the chasing of ‘surplus profits’ and technological rents are the most important source of these surplus profits. He too assumed that technological rentiership resulted from the need for firms to perpetually seek out new sources of innovation because it is hard to make new technologies permanently excludable in competitive markets.

### Frontiers of Techno-Economic Rentiership

From Marshall to Veblen to Schumpeter to Mandel, these thinkers assumed that economic rents were artificial advantages that would decline overtime, but political-economic transformations in the late 20^th^ and early 21^st^ centuries warrant a fresh look at their techno-economic underpinnings. For some the productivity gains of a long-touted shift to knowledge-driven networked markets are coming to fruition, with the World Economic Forum proclaiming a ‘Fourth Industrial Revolution’ (Schwab, 2016). But a burgeoning political economy literature has questioned such techno-optimism, instead stressing that recent market restructuring has been distinguished by the increasing prevalence of revenues from techno-economic ‘rentiership’ (Tretter, 2016; Andreucci et al. 2017; Birch, 2017a, 2017b; Rikap and Lundvall, 2020; Rikap, 2021a; Strauss, 2021). According to this latter interpretation, the white heat of the technological revolution is not only driving exponential digital productivity but also intensified and new forms of monopoly power, extractive relations, and distributional struggles. In an era of intellectual monopoly rents and active predation on innovation networks (Rikap, 2021b; Schwartz, 2020), it remains an open empirical question whether and how rentiership will dissipate under competition.

The resurgent interest in rentiership reflects the ever-increasing technoscientific nature of capitalism, requiring an analytical lens that takes techno-economic processes, practices and knowledge claims as core dynamics in political economy. As outlined above, the relationship between technoscientific innovation and capitalism has a long history, but this relationship is changing, and this can be seen in the emergent forms of techno-economic rentiership. The contributors to this Special Section build on the insights of diverse political-economic thinkers that have taken science, technology, and innovation as their focus in these discussions, stretching from more mainstream economics and management perspectives (e.g. Teece, 1986; Pisano, 1991) to an emerging critical mass of political-economic literature focusing on rentiership in contemporary, technoscientific capitalism (e.g. Zeller, 2008; Sayer, 2015, 2020; Schwartz, 2016; Srnicek, 2016; Standing, 2016; Tretter, 2016; Ward and Aalbers, 2016; Kay and Kenney-Lazar, 2017; Langley and Leyshon, 2017; Fields, 2019; Birch, 2020; Christophers, 2020; Rikap and Lundvall, 2020; Birch and Cochrane, 2021; Knuth, 2021; MacKenzie, 2021; Rikap, 2021a; Strauss, 2021).

Essential to these critical discussions are the ways that social actors pursue rentiership through their control over different techno-economic conditions, practices, and formations, often entailing intense struggles with other social actors. Understanding this means asking questions like, how are rents made? And by who? Here we highlight four examples of this techno-economic rentiership from the literature, presaging the papers in the Special Section, while acknowledging the diverse forms that techno-economic rentiership can take.

Firstly, while land rent is still a major component of wealth, it is being remade through techno-economic rentiership. One apt example is the remaking and reframing of land and the environment more generally as an ownable and alienable ‘environmental resource’ or ‘natural asset’ (Kay, 2017; Levidow, 2020; Ouma, 2020; Knuth, 2021). In his dissection of climate regulation, Felli (2014) outlines how legally created entitlements to pollute, such as carbon credits, represent a form of rentiership since carbon credits are fictitious commodities, in Karl Polanyi's terms, being a financial instrument resulting from government fiat, rather than a commodity produced for sale. According to Stratford (2020), these transformations raise important questions about who can capture these climate rents, how they are distributed, and how they are eventually eroded as part of a wider low-carbon transition (Knuth, 2021; Serna, 2021).

Secondly, finance is increasingly implicated in the transformation of natural resources, like land or water, into rent-bearing financial assets (Harvey, 1982, 2002; Haila, 1990; Fields, 2019; Purcell et al., 2020). Here, rentiership has a coordinative role in capitalism, as the search for rents drives specific speculative and productive investments (Haila, 1990) with the development of an attendant techno-economic apparatus (e.g. calculative devices, management practices, etc.) necessary to make these decisions. While this apparatus might imply a societal scale transformation, as the scholarship on financialization suggests, there is also a need to examine the everyday, individual contexts and formations. As Langley (2008) illustrates, finance has been extended into our personal lives through personal savings and borrowing, which is reflected in a range of emerging financial subjectivities (Kear, 2021). Other everyday examples include the financialization of housing (Fields, 2019) and the transformation of social reproduction into a financial asset (Strauss, 2021).

Thirdly, scholars like Zeller (2008), Sayer (2015), Schwartz (2016), and Rikap (2021a) emphasize the importance of the extension of intellectual property (IP) rights for simultaneously creating new revenue streams and new forms of extraction. This is most evident in the TRIPS articles of the World Trade Organization (WTO) that standardized IP rights, thereby requiring countries in the Global South, for example, to adopt these standards in exchange for access to markets (Zeller, 2008). According to Pagano and Rossi (2009), the global roll-out of IP rights and enforcement has led to a significant increase in fees and licencing income for countries in the Global North, especially to the USA. It has also enabled business organizations to outsource their supply chains by protecting their intellectual property (Rikap, 2021b). While some argue that the extension of IP rights has stymied innovation (Christophers, 2020), others argue that it has led to increased innovation through rentiership designed precisely to exploit the knowledge (and other) commons (Moulier Boutang, 2011; Vercellone, 2013; Birch and Cochrane, 2021).

Finally, as noted above, extending IP rights enables businesses to outsource their supply chains and operations, illustrating the ways that techno-economic changes in labour organization have enabled new forms of rentiership (Schwartz, 2020; Strauss, 2021). Obvious examples include the regulatory arbitrage evident in the gig economy, where platform firms benefit from externalizing both employment costs (Sadowski, 2020; Van Doorn and Badger, 2020) and asset investment and depreciation (Grabher 2020). However, less obvious examples include strategies of techno-economic scalability – via platforms, for example – underpinning large, monopsonist firms that reduce the capacity for labour bargaining and thereby reducing wages (Mihalyi and Szelenyi, 2019; Schwartz, 2020).

### Contributions to the Special Section

Each contribution to the Special Section explores a crucial new frontier of techno-economic rentiership. Schwartz offers a macro-historical overview connecting the emergence of intellectual monopolies with the position of labour in restructured value chains, while Rikap’s meso-scale analysis interrogates how the business model of such an intellectual monopoly (Amazon) centre on capturing dynamic innovation rents through predatory relations with its innovation network. Meanwhile, in two contrasting cases of wind farm developments in Mexico and the prevalence of algorithmic scoring in the USA, Alonso Serna and Kear’s contributions, respectively, show the distributional struggles that the capture of techno-economic rents gives rise to.

Lourdes Alonso Serna (2021) extends the analysis of land rentiership to new frontiers, unpacking how Mexico’s first large-scale wind energy development has created new opportunities for landowners to extract rents in the Isthmus of Tehuantepec, Oaxaca. Serna connects an analysis of how the techno-economic assemblages of wind power have unlocked additional land rents because of how those rents were created through environmental assetization. Here, she argues drawing on Andreucci et al. (2017) and Swyngedouw and Ward (forthcoming), the commodification of nature progresses through legally and technologically-mediated socio-political conflict over the formation of rent-bearing assets.

Mark Kear (2021) explores the distributional politics around ‘value grabbing’ of techno-economic rents as they are mediated by algorithmic scoring. Kear evokes E.P. Thompson’s ‘moral economy of the English Crowd’ and Sartre’s notion of ‘seriality’ as the formation of an unconscious, ‘postsocial’ collective unified by an object (e.g. an algorithm). He then explores social claim making in response to algorithmic scoring as one of subjectification within a dialectic of both resistance and acquiescence to data extraction. He suggests that the emerging moral economy involves novel distributional claims in response to the algorithms’ facilitation of rent extraction and concurrent new forms of differentiation and marginalization.

Cecilia Rikap’s (2021b) contribution offers a close reading of the business model of one intellectual monopoly, Amazon. Technorents arise from the artificial scarcity produced by IP. Rikap observes how intellectual monopolies rely on both legal powers to exclude and the capacity to capture and exploit innovation networks; she calls this the gathering ‘dynamic innovation rents’ (Durand and Milberg, 2020). Utilizing patent data to indicate the extent of Amazon’s predation on its innovation network, Rikap argues that this enables extension across multiple fronts of rentiership because Amazon’s centralization and use of intangible assets enables greater leverage to financialize their revenues.

Finally, Schwartz (2020) traces how OECD economies’ value chains shifted from dualistic models under Fordism to today’s ‘vertically disintegrated’ value chains dominated by those firms able to marshal monopoly rents through intellectual property rights. The resultant three-tier economy value chain structure has seen profitable IPR firms exercise de facto, without *de jure*, control over companies lower in the value chain. Lower down the hierarchy are intermediate companies that retain decent profitability through specialized production processes and tacit knowledge, and low-profit firms whose margins lie in the (hyper-)exploitation of their labour force. Schwartz contextualizes how the contemporary centrality of technorents are underpinned by changes to the organizational structure of OECD economies, which have driven social and spatial inequalities.

## References

[bibr1-10245294221082243] Andreucci D Garcia-Lamarca M Wedekind J Swyngedouw E (2017) “Value grabbing”? A political ecology of rent. Capitalism Nature Socialism 28(3): 28–47.

[bibr2-10245294221082243] Birch K (2017a) Financing ttechnoscience: finance, aassetization and rrentiership. In: Tyfield D Lave R Randalls S Thorpe C. (eds) The Routledge Handbook of the Political Economy of Science. London, UK: Routledge, 169–181

[bibr3-10245294221082243] Birch K (2017b) Towards a theory of rentiership. Dialogues in Human Geography 7(1): 109–111.

[bibr4-10245294221082243] Birch K (2020) Technoscience rent: toward a theory of rentiership for technoscientific capitalism, science. Technology & Human Values. 45(1): 3–33.

[bibr5-10245294221082243] Birch K Cochrane T (2021) Big tech: four emerging forms of digital rentiership. Science as Culture. 31(1): 44–58.

[bibr6-10245294221082243] Birch K Cochrane DT Ward C (2021) Data as asset? The measurement, governance, and valuation of digital personal data by Big Tech. Big Data & Society 8(1): 1–15.

[bibr7-10245294221082243] Christophers B (2020) Rentier Capitalism. London, UK: Verso.

[bibr8-10245294221082243] Durand C Milberg W (2020) Intellectual monopoly in global value chains. Review of International Political Economy 27(2): 404–429.

[bibr9-10245294221082243] Felli R (2014) On Climate Rent. Historical Materialism 22(3-4): 251–280.

[bibr10-10245294221082243] Frase P (2016) Four Futures: Life After Capitalism. London, UK: Verso.

[bibr11-10245294221082243] Haila A (1990) The theory of land rent at the crossroads. Environment and Planning D: Society and Space 8(3): 275–296.

[bibr12-10245294221082243] Haila A (2016) Urban Land Rent. Malden, MA: Wiley-Blackwell

[bibr13-10245294221082243] Harvey D (1982) The Limits to Capital. Oxford, UK: Blackwell.

[bibr14-10245294221082243] Harvey D (2002) The art of rent: globalization, monopoly and the commodification of culture. Socialist Register 38: 93–110.

[bibr15-10245294221082243] Kay K (2017) Rural rentierism and the financial enclosure of maine’s open lands tradition. Annals of the American Association of Geographers 107(6): 1–17

[bibr16-10245294221082243] Kay K Kenney-Lazar M (2017) Value in capitalist natures: an emerging framework. Dialogues in Human Geography 7(3): 295–309.

[bibr17-10245294221082243] Kear M (2021) The moral economy of the algorithmic crowd: possessive collectivism and techno-economic rentiership. Competition & Change. DOI: 10.1177/1024529421990496

[bibr18-10245294221082243] Knuth S (2021) Rentiers of the low-carbon economy? Renewable energy’s extractive fiscal geographies. Environment & Planning A: Economy and Space. DOI: 10.1177/0308518X211062601

[bibr19-10245294221082243] Komljenovic J (2021) The rise of education rentiers: digital platforms, digital data and rents. Learning, Media and Technology 46(3): 320–332.

[bibr20-10245294221082243] Langley P (2008) The Everyday Life of Global Finance. Oxford, UK: Oxford University Press

[bibr21-10245294221082243] Langley P Leyshon A (2017) Platform capitalism: the intermediation and capitalisation of digital economic circulation. Finance and Society 3(1): 11–31.

[bibr22-10245294221082243] MacKenzie D (2021) Trading at the Speed of Light: How Ultrafast Algorithms Are Transforming Financial Markets. Princeton, NJ: Princeton University Press

[bibr23-10245294221082243] Mandel E (1975) Late Captialism. London, UK: NLB

[bibr24-10245294221082243] Marshall A (1890, 1920) Principles of Economics. London, UK: Macmillan & Co Ltd

[bibr25-10245294221082243] Marx K . (1894). Capital: Part III, New York, NY: International Publishers, available at: https://www.marxists.org/archive/marx/works/1894-c3/.

[bibr26-10245294221082243] Mazzucato M (2018) The Value of Everything. London, UK: Allen Lane.

[bibr27-10245294221082243] McGoey L (2017) The elusive rentier rich: piketty’s data battles and the power of absent evidence. Science, Technology & Human Values 42(2): 257–279.

[bibr28-10245294221082243] McGoey L (2021) Hiding the rentier elephant in plain sight: the epistemology of vanishing rent. Sociologica 15(2): 75–93.

[bibr29-10245294221082243] Moulier Boutang Y (2011) Cognitive Capitalism. Cambridge MA. Polity

[bibr30-10245294221082243] Ouma S (2020) Farming as a Financial Asset. Newcastle, UK: Agenda Publishing

[bibr31-10245294221082243] Pagano H Rossi M (2009) The crash of the knowledge economy. Cambridge Journal of Economics 33(4): 665–683.

[bibr32-10245294221082243] Piketty T (2014) Capital in the Twenty-First Century. Cambridge, MA. Belknap Press

[bibr33-10245294221082243] Pisano G (1991) The governance of innovation: vertical integration and collaborative arrangements in the biotechnology industry. Research Policy 20(3): 237–249

[bibr34-10245294221082243] Ricardo D . 1817 (2001). On the Principles of Political Economy and Taxation, Kitchener, Canada: Batoche Books

[bibr35-10245294221082243] Rikap C Lundvall B-A . 2020. Big tech, knowledge predation and the implications for development. Innovation and Development. DOI: 10.1080/2157930X.2020.1855825

[bibr36-10245294221082243] Rikap C (2021a) Capitalism, Power and Innovation: Intellectual Monopoly Capitalism Uncovered. London, UK: Routledge.

[bibr37-10245294221082243] Rikap C (2021b) Amazon: A story of accumulation through intellectual rentiership and predation. Competition & Change. DOI: 10.1177/1024529420932418

[bibr38-10245294221082243] Sadowski J (2020) The internet of landlords: digital platforms and new mechanisms of rentier capitalism. Antipode 52(2): 562–580

[bibr39-10245294221082243] Sayer A (2015) Why We Can’t Afford the Rich. Bristol, England. Policy Press.

[bibr40-10245294221082243] Schumpeter J (1950) Capitalism, Socialism and Democracy. New York, NY: Harper Perennial

[bibr41-10245294221082243] Schwartz HM (2016) Wealth and secular stagnation: The role of industrial organization and intellectual property rights. RSF: The Russell Sage Foundation Journal of the Social Sciences 2(2): 226–249.

[bibr42-10245294221082243] Schwartz HM (2020) Intellectual property, technorents and the labour share of production. Competition & Change. DOI: 10.1177/1024529420968221

[bibr43-10245294221082243] Serna LA (2021) Land grabbing or value grabbing? Land rent and wind energy in the Isthmus of Tehuantepec, Oaxaca. Competition & Change. DOI: 10.1177/10245294211018966

[bibr44-10245294221082243] Smith A (1776) An Inquiry into the Nature and Causes of the Wealth of Nations. available at: https://www.econlib.org/library/Smith/smWN.html

[bibr45-10245294221082243] Srnicek N (2016) Platform Capitalism. Cambridge, MA: Polity Press

[bibr46-10245294221082243] Standing G (2016) The Corruption of Capitalism. London, UK: Biteback publishing.

[bibr47-10245294221082243] Stiglitz J (2012) The Price of Inequality. New York, NY. W.W. Norton & Company

[bibr48-10245294221082243] Stratford B (2020) The threat of rent extraction in a resource-constrained future. Ecological Economics 169: 1–11.

[bibr49-10245294221082243] Strauss K . (2021). Beyond crisis? Using rent theory to understand the restructuring of publicly funded seniors’ care in British Columbia, Canada. Environment & Planning A. DOI: 10.1177/0308518X20983152 PMC10555532 37810991

[bibr50-10245294221082243] Storper M (1997) The Regional World, Territorial Development in a Global Economy. New York, NY: Guilford Press

[bibr51-10245294221082243] Teece D (1986) Profiting from technological innovation: implications for Integration, Collaboration, Licensing and Public Policy. Research Policy 15(6): 285–305.

[bibr52-10245294221082243] Veblen T . (1904). The Theory of Business Enterprise New York, NY: Charles Scribner's Sons, available at: https://brocku.ca/MeadProject/Veblen/Veblen_1904/Veblen_1904_03.html.

[bibr53-10245294221082243] Vercellone C (2013) The becoming rent of profit? The new articulation of wage, rent and profit. Knowledge Cultures 1(2): 194–207.

[bibr54-10245294221082243] Wark M (2019) Capital is Dead: Is This Something Worse? London, UK: Verso

[bibr55-10245294221082243] Ward C Aalbers M (2016) Virtual special issue editorial essay ‘The shitty rent business’: what’s the point of land rent theory? Urban Studies 53(9): 1760–1783

[bibr56-10245294221082243] Zacares J (2021) Euphoria of the Rentier? New Left Review 129 May/June.

[bibr57-10245294221082243] Zeller C (2008) From the gene to the globe: extracting rents based on intellectual property monopolies. Review of International Political Economy 15(1): 86–115.

